# The effects of PTPN2 loss on cell signalling and clinical outcome in relation to breast cancer subtype

**DOI:** 10.1007/s00432-019-02918-y

**Published:** 2019-04-25

**Authors:** Cynthia Veenstra, Elin Karlsson, Sanam Mirwani Mirwani, Bo Nordenskjöld, Tommy Fornander, Gizeh Pérez-Tenorio, Olle Stål

**Affiliations:** 1 Division of Clinical Sciences, Department of Clinical and Experimental Medicine and Department of Oncology, Faculty of Health Sciences, Linköping University, 581 85 Linköping, Sweden; 2 Department of Oncology-Pathology, Karolinska University Hospital and Karolinska Institute, Stockholm, Sweden

**Keywords:** PTPN2, TCPTP, ddPCR, IHC, Breast cancer, Akt, Met, HGF

## Abstract

**Purpose:**

The protein tyrosine phosphatase PTPN2 dephosphorylates several tyrosine kinases in cancer-related signalling pathways and is thought to be a tumour suppressor. As PTPN2 is not frequently studied in breast cancer, we aimed to explore the role of PTPN2 and the effects of its loss in breast cancer.

**Methods:**

Protein expression and gene copy number of PTPN2 were analysed in a cohort of pre-menopausal breast cancer patients with immunohistochemistry and droplet digital PCR, respectively. PTPN2 was knocked down in three cell lines, representing different breast cancer subtypes, with siRNA transfection. Several proteins related to PTPN2 were analysed with Western blot.

**Results:**

Low PTPN2 protein expression was found in 50.2% of the tumours (110/219), gene copy loss in 15.4% (33/214). Low protein expression was associated with a higher relapse rate in patients with Luminal A and HER2-positive tumours, but not triple-negative tumours. In vitro studies further suggested a subtype-specific role of PTPN2. Knockdown of PTPN2 had no effect on the triple-negative cell line, whilst knockdown in MCF7 inhibited phosphorylation of Met and promoted that of Akt. Knockdown in SKBR3 led to increased Met phosphorylation and decreased Erk phosphorylation as well as EGF-mediated STAT3 activation.

**Conclusion:**

We confirm previous studies showing that the PTPN2 protein is lost in half of the breast cancer cases and gene deletion occurs in 15–18% of the cases. Furthermore, the results suggest that the role of PTPN2 is subtype-related and should be further investigated to assess how this could affect breast cancer prognosis and treatment response.

## Introduction

Phosphorylation of tyrosine kinases is an important mechanism in cellular signalling driving tumourigenesis. Protein tyrosine phosphatases (PTPs) regulate phosphorylation by removing phosphoryl groups and bringing tyrosine kinases back to their original state. PTPN2, also known as TCPTP, is an intracellular non-transmembrane phosphatase that is ubiquitously expressed (Cool et al. 1989). There are two main isoforms of PTPN2, namely the 48 kDa variant (TC48) and the 45 kDa variant (TC45) (Cool et al. 1989; Champion-Arnaud et al. 1991). A third, less common, isoform is the 41 kDa (TC41) isoform (Bussieres-Marmen et al. 2014). TC48 is localised to the endoplasmic reticulum, whilst TC45 resides in the nucleus with the possibility of translocating to the cytoplasm upon stimuli, like growth factors or cellular stress (Tiganis et al. 1998; Lam et al. 2001). PTPN2 has been proposed as a tumour suppressor, following several deletion and overexpression studies in haematological and solid malignancies (Kleppe et al. 2010; Karlsson et al. 2015; Shields et al. 2013; Hoshida et al. 2008; Lee et al. 2009).

Breast cancer is a heterogeneous disease, subdivided into several subtypes. The least aggressive subtype is known as Luminal A and is characterised as oestrogen receptor (ER) and progesterone receptor (PR)-positive, human epidermal growth factor receptor-2 (HER2)-negative and usually Nottingham grade (NHG) 1 or 2. The Luminal B subtype is further divided into B1 and B2. Luminal B1 is ER-positive, HER2-negative, and NHG 3, whilst Luminal B2 is positive for ER and HER2. Tumours negative for ER and PR, and positive for HER2 are called HER2-like tumours. Triple-negative breast cancer tumours are distinguished by their lack of the three markers. The three last-mentioned subtypes are mostly NHG 2/3 and the patient outcome is often poor (Goldhirsch et al. 2011).

While PTPN2 is commonly found deleted in non-Hodgkin lymphoma and T-cell acute lymphoblastic leukaemia, few studies exist on the role of PTPN2 in breast cancer (Kleppe et al. 2010, 2011). The PTPN2 protein has been shown to be lost in ER-negative breast cancer, more so in triple-negative breast cancers (TNBC) (Shields et al. 2013) and we have previously found that the protein is expressed at low levels in 53.3% of the tumours in a cohort of low-risk breast cancer patients (Karlsson et al. 2018). *PTPN2* gene copy loss was reported in 16% and 18% in a high-risk post-menopausal breast cancer cohort and low risk, respectively. Loss was correlated with poor patient outcome in the high-risk cohort (Karlsson et al. 2015, 2018). Various substrates of PTPN2 play important roles in the genesis and progression of breast cancer amongst others the epidermal growth factor receptor (EGFR), STAT3, and proposedly the Met receptor (Sangwan et al. 2008; Tiganis et al. 1998, 1999; Yamamoto et al. 2002). In this study, the role of PTPN2 was explored in the different subtypes of breast cancer in both a subset of pre-menopausal breast cancer patients and cell lines.

## Materials and methods

### Patient material

**Fig. 1:**
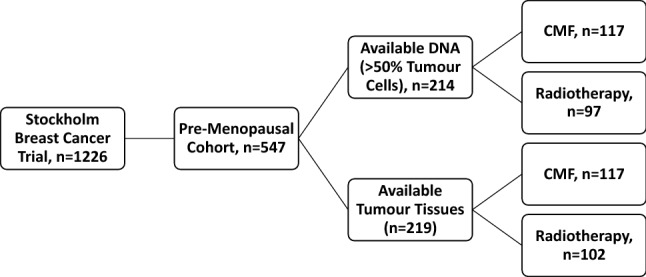
Patient distribution throughout the pre-menopausal breast cancer patient cohort. Patients were randomised to receive either radiotherapy or chemotherapy. *CMF* cyclophosphamide, methotrexate, and 5-fluoruocil

Between 1976 and 1990, the Stockholm breast cancer trial recruited a total of 1226 pre- and post-menopausal patients with tumours larger than 30 mm and/or positive lymph nodes in a randomised trial comparing 46 Gy of loco-regional post-operative radiotherapy with 12 courses of Milan-type CMF adjuvant chemotherapy (Bonadonna et al. 1976). There were 547 pre-menopausal patients included in the trial (Rutqvist and Johansson 2006). Tumour tissues obtained from surgery were formalin-fixed, paraffin-embedded (FFPE) and stored at room temperature until usage. Genomic DNA was previously extracted from FFPE tumour tissues using QIAamp DNA FFPE Tissue Kit (Qiagen, Hilden, Germany) (Veenstra et al. 2016). Tumour tissues were available from 219 of the pre-menopausal patients and DNA extracted from the FFPE tissues was available from 214 (Fig. 1). Retrospective studies on this tumour material were approved by the ethical committee at Karolinska Institute in Stockholm, Sweden. The patient and treatment characteristics are displayed in Table 1. ER status was obtained by isoelectric focusing, with a threshold of 0.05 fmol/µg DNA. HER2 overexpression was established by immunohistochemical analysis, per the Herceptest Guidelines for membrane staining (Dako Agilent, Santa Clara, CA, USA). Phospho-Akt-S473 (pAkt), pMet-Y1349 (pMet), and HGF have been previously analysed by immunohistochemistry (IHC) (Veenstra et al. 2016).

**Table 1:** Patient characteristics and clinicopathological parameters in association with PTPN2 protein expression and PTPN2 copy loss

	Total (tissue)	PTPN2 expression		*p* value	Total (DNA)	*PTPN2* copy loss		*p* value
	*n* (%)	Low	High		*n* (%)	Deletion	≥ 2 copies	
		*n* (%)	*n* (%)			*n* (%)	*n* (%)	
Total	219	110 (50.2)	109 (49.8)		214	33 (15.4)	181 (84.6)	
Lymph node status								
0	28 (12.8)	14 (50.0)	14 (50.0)	0.998	27 (12.6)	2 (7.4)	25 (92.6)	0.356
1–3	121 (55.3)	61 (50.4)	60 (49.6)		116 (54.2)	21 (18.1)	95 (81.9)	
> 3	70 (32.0)	35 (50.0)	35 (50.0)		71 (33.2)	10 (14.1)	61 (85.9)	
Tumour size (mm)								
≤ 20	81 (37)	40 (49.4)	41 (50.6)	0.846	83 (38.8)	11 (13.3)	72 (86.7)	0.502
> 20	132 (60.3)	67 (50.8)	65 (49.2)		126 (58.9)	21 (16.7)	105 (83.3)	
Unavailable	6 (2.7)				5 (2.3)			
NHG								
1	47 (21.5)	20 (42.6)	27 (57.4)	0.573	50 (23.4)	6 (12.0)	44 (88.0)	0.679
2	112 (51.1)	57 (50.9)	55 (49.1)		109 (50.9)	19 (17.4)	90 (82.6)	
3	52 (23.7)	27 (51.9)	25 (48.4)		46 (21.5)	7 (15.2)	39 (84.8)	
Unavailable	8 (3.7)				9 (4.2)			
Adjuvant treatment								
CMF	117 (53.4)	54 (46.2)	63 (53.8)	0.197	117 (54.7)	15 (12.8)	102 (87.2)	0.247
RT	102 (46.6)	56 (54.9)	46 (45.1)		97 (45.3)	18 (18.6)	79 (81.4)	
ER status								
Negative*	59 (26.9)	35 (59.3)	24 (40.7)	**0.037**	55 (25.7)	5 (9.1)	50 (90.9)	0.131
Positive^†^	139 (63.5)	60 (43.2)	79 (56.8)		141 (65.9)	25 (17.7)	116 (82.3)	
Unavailable	21 (9.6)				18 (8.4)			
HER2 status								
Negative	183 (83.6)	92 (50.3)	91 (49.7)	0.900	180 (84.1)	28 (15.6)	152 (84.4)	0.953
Positive	35 (16)	18 (51.4)	17 (48.6)		33 (15.4)	5 (15.2)	28 (84.8)	
Unavailable	1 (0.4)				1 (0.5)			
pAkt cytoplasm								
Negative	113 (51.6)	70 (61.9)	43 (38.1)	**<** **0.001**	106 (49.5)	21 (63.6)	85 (49.7)	0.101
Positive	104 (47.5)	38 (36.5)	66 (63.5)		98 (45.8)	12 (36.4)	86 (50.3)	
Unavailable	2 (0.9)				10 (4.7)			
pAkt N≥C								
No	149 (68)	64 (43.0)	85 (57.0)	**0.003**	142 (66.4)	20 (14.1)	122 (85.9)	0.219
Yes	68 (31.1)	44 (65.7)	23 (34.3)		62 (28.9)	13 (21.0)	49 (79.0)	
Unavailable	2 (0.9)				10 (4.7)			
pMet membrane								
Negative	158 (72.1)	94 (59.5)	64 (40.5)	**<** **0.001**	147 (68.7)	22 (15.0)	125 (85.0)	0.472
Positive	55 (25.1)	16 (29.1)	39 (70.9)		52 (24.3)	10 (19.2)	42 (80.8)	
Unavailable	6 (2.8)				15 (7)			
pMet cytoplasm								
Negative	100 (45.7)	75 (75.0)	25 (25.0)	**<** **0.001**	90 (42.1)	15 (16.7)	75 (83.3)	0.838
Positive	113 (51.6)	35 (31.0)	78 (69.0)		109 (50.9)	17 (15.6)	92 (84.4)	
Unavailable	6 (2.7)				15 (7)			
HGF stroma								
Negative	102 (46.6)	55 (53.9)	47 (46.1)	0.243	98 (45.8)	14 (14.3)	84 (85.7)	0.440
Positive	109 (49.8)	50 (45.9)	59 (54.1)		98 (45.8)	18 (18.4)	80 (81.6)	
Unavailable	8 (3.6)				18 (8.4)			
HGF cytoplasm								
Negative	108 (49.3)	65 (60.2)	43 (39.8)	**0.003**	104 (48.6)	17 (16.3)	87 (83.7)	0.967
Positive	104 (47.4)	41 (39.4)	63 (60.6)		93 (43.5)	15 (16.1)	78 (83.9)	
Unavailable	5 (2.3)				17 (7.9)			
Breast cancer subtype								
Luminal A	107 (48.0)	49 (45.8)	58 (54.2)	0.412	109 (50.9)	18 (16.5)	91 (83.5)	0.337
Luminal B1	13 (5.9)	4 (30.8)	9 (69.2)	0.152	14 (6.5)	3 (21.4)	11 (78.6)	0.483
Luminal B2	14 (6.4)	5 (35.7)	9 (64.3)	0.261	14 (6.5)	3 (21.4)	11 (78.6)	0.549
HER2	16 (7.3)	10 (62.5)	6 (37.5)	0.299	14 (6.5)	2 (14.3)	12 (85.7)	0.612
TNBC	43 (19.6)	25 (58.1)	18 (41.9)	0.145	40 (18.7)	3 (7.5)	37 (92.5)	0.137
Unavailable	26 (12.8)				23 (10.7)			

*ER* oestrogen receptor, *NHG* Nottingham Grade, *N ≥ C* nuclear expression equal to or bigger than cytoplasmic expression, *TNBC* triple-negative breast cancer

*p* values printed in bold are considered significant

*< 0.05 fmol/µg DNA, ^†^≥ 0.05 fmol/µg DNA

### Tissue microarray

Tissue microarrays (TMAs) of the available tumour tissues were manufactured as follows: representative tumour tissue blocks were used as donor blocks, sections from these blocks were stained with haematoxylin and eosin after which three biologically representative regions were selected for all tumour samples. Three tissue cores of 0.8 mm in diameter were taken from these regions and re-embedded in paraffin blocks. The blocks were cut into 5 µM sections and placed on frost-coated microscope slides. The sections were covered with a layer of paraffin upon cutting and stored at 4 °C.

### Immunohistochemistry

TMA sections were cleared from the paraffin layer by upright incubation at 60 °C. Sections were further deparaffinised, rehydrated, and antigen-retrieved using the DAKO PT Module (PT Link, Dako Agilent) with DAKO PT Low pH Buffer (Envision FLEX target retrieval solution low, Dako Agilent). The sections were incubated for 10 min with serum-free protein block to reduce unspecific binding (Spring Bioscience, Fremont, CA, USA). The sections were incubated overnight at 4 °C with a PTPN2 antibody recognising both main isoforms (Proteintech, Rosemont, IL, USA; 11214-1-AP, diluted 1:40) prior to 30 min’ incubation at room temperature with an anti-rabbit secondary antibody (EnVision™, Dako Agilent). The arrays were then coloured with 3′-diaminobenzidine tetrahydrochloride (DAB/H_2_O_2_) solution and counterstained with Mayer’s haematoxylin (Fluka Analytical, Sigma-Aldrich, St. Louis, MO, USA). Dehydration was established using a series of ethanol dilutions. Whole-slide images were obtained with Aperio ScanScope AT (Leica Microsystems, Wetzler, Germany). Grading was performed by three independent investigators (CV, EK, and SMM) and cytoplasmic staining of tumour cells was graded as negative, weak, intermediate, or strong. PTPN2 protein staining could be successfully evaluated in 219 tumours. For analyses, groups were dichotomised into low (negative-to-weak) and high (intermediate-to-strong). Protein specificity of the PTPN2 antibody was previously validated in our lab (Karlsson et al. 2018).

### Droplet digital PCR

*PTPN2* copy number variations (CNV) were measured with droplet digital PCR (ddPCR). Primers and probe against *PTPN2* exon 10 (accession number: NM_080422.2) were designed using Primer Expression v1.5a (Applied Biosystems, Carlsbad, CA, USA). Sequences were as follows: forward primer: 5′-AAGCCCACTCCGGAAACTAAA-3′, reversed primer: 5′-AAACAAACAACTGTGAGGCAATCTA-3′, probe: 5′-TGAGGCTCGCTAACC-3′. The resulting product had a total amplicon length of 65 nt. The annealing temperature was 64.2 °C. *AP3B1* (dHsaCP2500348; Bio-Rad, Hercules, CA, USA) was chosen as the reference gene as previously described, as well as the followed protocol (Veenstra et al. 2016). CNV of *PTPN2* could be successfully assessed in 214 tumours.

### Cell lines and growth factor treatment

The breast cancer cell lines MCF7, MDA-MB-468, and SKBR3 cells were purchased from the American Type Culture Collection (ATCC, Manassas, VA, USA), the authenticity of the cell lines was validated prior purchase, using short-tandem repeat (STR) profiling. MCF7, representing Luminal A disease, and MDA-MB-468, representing TNBC, were cultured in DMEM high glucose media supplemented with 10% foetal bovine serum and 0.5% l-glutamine (complete medium) (Gibco, Invitrogen, Carlsbad, CA, USA). SKBR3, representing HER2-like breast cancer, was cultured in DMEM media supplemented with 10% foetal bovine serum and 0.5% l-glutamine (complete medium) (Gibco). Experiments were performed between passage 5 AND 30. The cells were starved 24 h prior to growth factor treatment in medium containing 0.5% FBS (low-serum medium). Cells were treated with 30 ng/mL human EGF with carrier [Cell Signaling Technology (CST), Beverly, MA, USA] and/or 50 ng/mL recombinant human HGF diluted in PBS containing 0.5% BSA (CST).

### siRNA transfection

Cells were seeded in six-well culture plates in complete medium at 2 × 10^5^ cells/mL shortly before transfection and incubated under standard conditions until transfection. The cells were transfected with 10 nM scrambled siRNA (AllStars Negative Control siRNA, Qiagen) or 10 nM siRNA targeted against both main isoforms of *PTPN2* (siRNA_15 Qiagen) using HiPerFect reagent (Qiagen), following the manufacturer’s fast forward protocol. After 48 h, cells were used for follow-up experiments. A positive control siRNA (AllStars Hs Cell Death Control, Qiagen), knocking down ubiquitous human cell survival genes, was included in every experiment. All positive controls performed resulted in > 90% cell death as visualised by light microscopy. Unspecific effects of transfection reagents were discarded by mock-transfecting the cells during the optimisation of the experiments. Knockdown control was performed using Western blot and gene expression analysis (at least 80% knockdown per experiment; results not shown).

### Western blot analysis

The cells were harvested in RIPA lysis buffer containing protease inhibitor cocktail (cOmplete, Roche, Basil, Switzerland) and phosphatase inhibitor (PhosSTOP, Roche), lysates were snap-frozen and stored at − 70 °C until usage. Twenty-five µg protein samples were loaded on a pre-cast Mini Protean TGX 4–15% gradient gels (Bio-Rad) and separated by electrophoresis. The proteins were then transferred to a membrane using the Trans-Blot Turbo Transfer System (Bio-Rad). The membranes were blocked for 1 h at room temperature in blocking buffer (TBST with 5% milk powder or BSA). The membranes were incubated overnight at 4 °C with primary antibodies, after thorough washing, the membranes were incubated for 1 h at room temperature with appropriate HRP-conjugated secondary antibodies (Dako Agilent). Primary antibodies used: phospho-Met Y1349 (pMet, 1:1000, Abcam, Cambridge, UK), pEGFR Y1068 (pEGFR, 1:1000, CST), pAkt S473 (pAkt, 1:1000, CST), pErk 1/2 Thr202/Y204 (pErk, 1:1000, CST), p-STAT3 Y705 (pSTAT3, 1:1000, CST), PTPN2 TC45 (1:1000, CST), and GAPDH-HRP conjugated (1:5000, Abcam).

### Statistical analyses

Relationships between factors were assessed using Pearson’s Chi squared test. Survival of patients in different groups was calculated using Kaplan–Meier and the Mantel–Cox test estimated the statistical differences. Hazard ratio (HR) was calculated with Cox proportional hazards regression and reported with 95% confidence intervals (CI). Distant recurrence-free survival (DRFS) was defined as the period of time passed between the diagnosis of the primary tumour and the distant recurrence. Loco-regional recurrence-free survival was defined likewise but for local–regional recurrence. A multivariate Cox model was carried out to assess the interaction between benefit from radiotherapy versus chemotherapy (CMF) and PTPN2 protein expression. As described previously, in this cohort of patients the subtypes were defined by ER-status, HER2-status, and NHG; Ki67 staining was not available (Veenstra et al. 2016). For survival analysis, overexpression of HER2, rather than the HER2-like subtype, was used, as there were few patients in the HER2-like subtype group. Statistical analyses were performed with IBM SPSS for Windows, Version 23 (IBM Corp, Armonk, NY, USA). The criterion for statistical significance was *p* ≤ 0.05.

## Results

### PTPN2 copy number variations and protein expression

To determine the expression levels of PTPN2, IHC was carried out with an antibody recognising both main isoforms of PTPN2 (TC45 and TC48). Expression was primarily localised to the cytoplasm and high expression (intermediate to strong staining) was found in 49.8% (109/219) of the tumours, low expression in 50.2% (110/219) of the cases (Table 1). CNV was analysed by ddPCR in DNA extracted from the tumours. *PTPN2* copy loss was detected in 15.4% (33/214) and two or more copies were found in 84.6% (181/214) (Table 1). Copy numbers and protein expression did not correlate with each other (*p* = 0.7).

### PTPN2 in relation to clinicopathological characteristics

Whilst *PTPN2* copy loss did not correlate to any of the clinicopathological parameters, low PTPN2 protein expression was found to be correlated with ER-negative tumours and pAkt status in the cytoplasm. When pAkt expression was mainly localised to the nucleus (pAkt N≥C), it correlated with low PTPN2 expression.

The proposed PTPN2 substrate Met was found to be associated with PTPN2, membranous and cytoplasmic phosphorylated Met were more often positive in PTPN2-positive tumours, which was specifically apparent in the Luminal A subtype (cytoplasmic pMet: *p* < 0.005 and membranous pMet: *p* = 0.038). Cytoplasmic HGF, Met’s ligand, correlated with PTPN2 expression. None of the breast cancer subtypes showed a direct correlation with PTPN2 staining or copy loss. All associations are listed in Table 1.

### PTPN2 is correlated with poor outcome

**Fig. 2:**
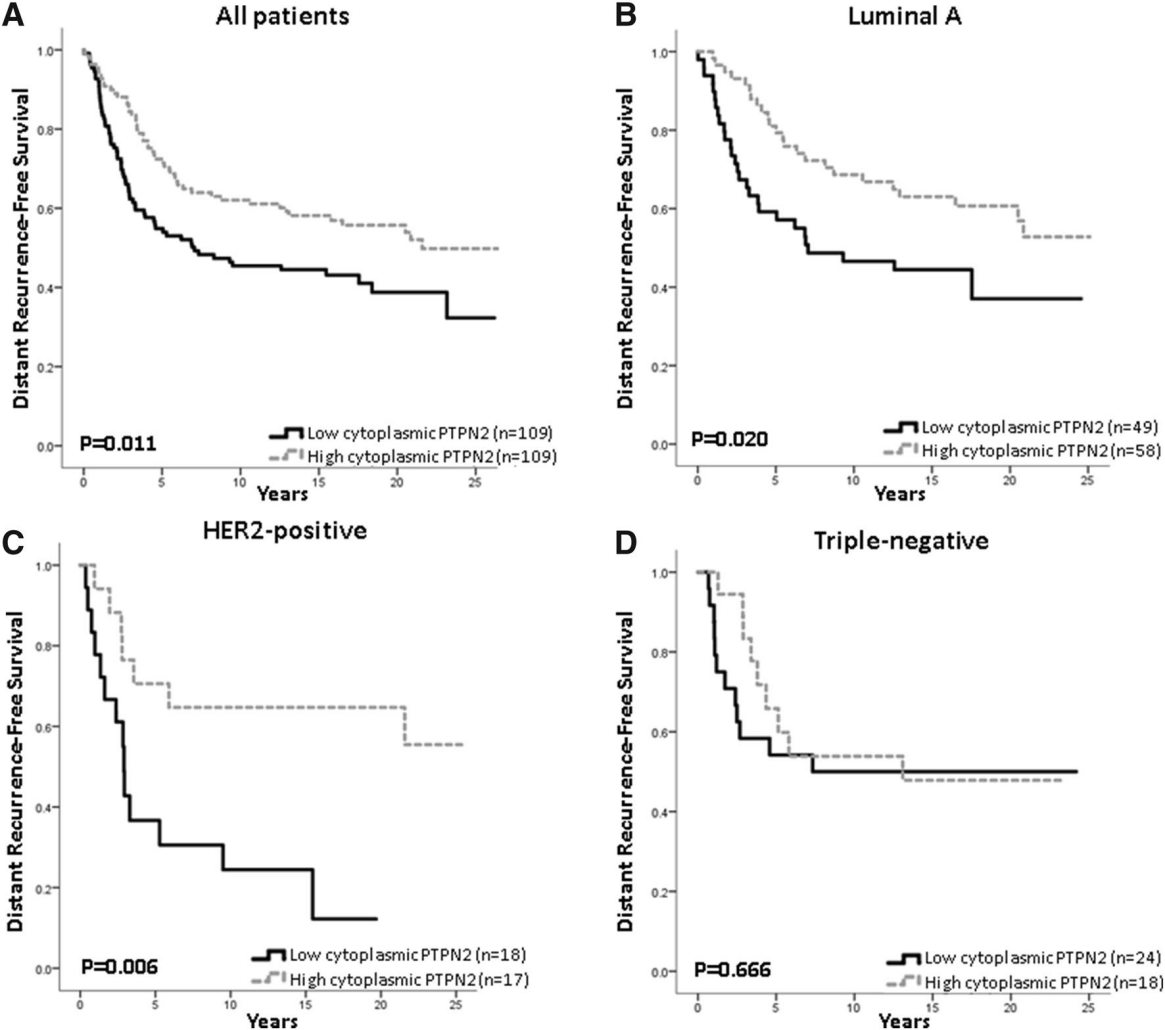
Distant recurrence-free survival analyses of patients with tumours showing low or high PTPN2 protein expression, in **a** all patients (high vs low HR = 0.62; 95% CI 0.43–0.90, *p* = 0.012), **b** patients with Luminal A disease (HR = 0.53; 95% CI 0.30–0.91, *p* = 0.022), **c** patients with HER2 overexpressing tumours (HR = 0.28; 95% CI 0.11–0.74, *p* = 0.010), and **d** patients with triple-negative disease (HR = 0.83; 95% CI 0.35–1.97; *p* = 0.67)

**Fig. 3:**
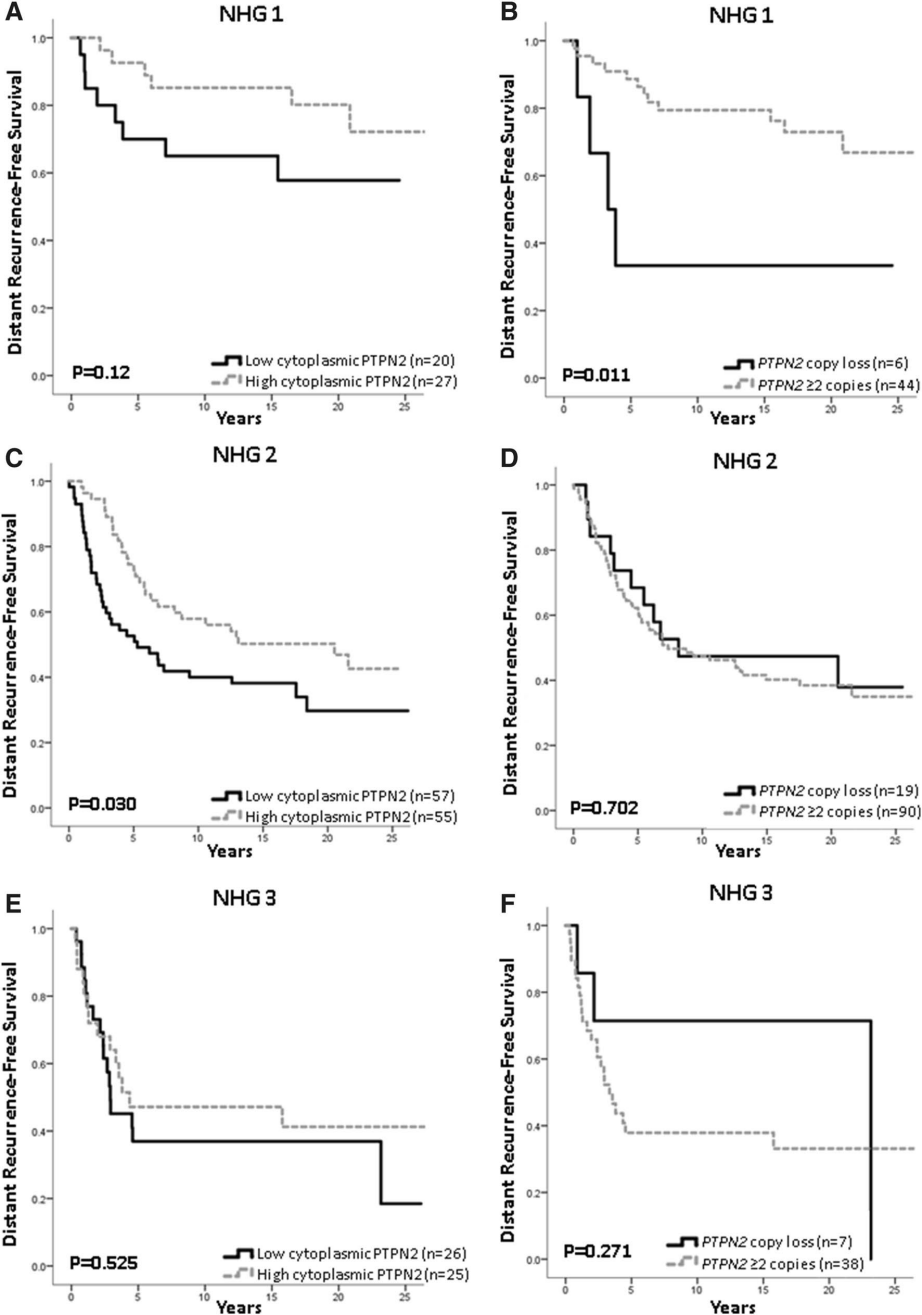
Survival analyses in relation to protein or copy loss and NHG status. Survival in patients with tumours with **a** protein loss with NHG 1 tumours, (high vs low) HR = 0.46; 95% CI 0.15–1.25, *p* = 0.12; **b***PTPN2* gene copy loss with NHG 1 tumours, (copy loss vs ≥ 2 copies) HR = 3.93; 95% CI 1.25–12.3, *p* = 0.019; **c** protein loss with NHG 2 tumours, HR = 0.59; 95% CI 0.36–0.96, *p* = 0.032; **d** gene copy loss in NHG 2 tumours, HR = 0.88; 95% CI 0.46–1.68, *p* = 0.70; **e** protein loss in NHG 3 tumours, HR = 0.79; 95% CI 0.39–1.62, *p* = 0.53; **f** copy loss with NHG 3 tumours, HR = 0.51; 95% CI 0.13–1.72, *p* = 0.28

Low PTPN2 protein expression was associated with a higher risk for distant recurrence (Fig. 2a), this was even more apparent in the Luminal A subtype and tumours with HER2 overexpression, but not in the triple-negative subtype (Fig. 2b–d). *PTPN2* gene copy number did not have prognostic value in relation to all patients or subtypes (copy loss vs ≥ 2 copies, all patients: HR = 1.15; 95% CI 0.70–1.88, *p* = 0.58; Luminal A: HR = 1.44; 95% CI 0.75–2.8, *p* = 0.28; HER2 positive: HR = 0.19; 95% CI 0.03–1.39, *p* = 0.1; TNBC: HR = 0.53; 0.07–3.97, *p* = 0.538). Protein loss was found to be related to worse survival in patients with tumours with NHG 2, with a similar trend for NHG 1, whilst *PTPN2* copy loss was associated with worse survival in patients with tumours with NHG 1, neither was associated with DRFS at a high grade (Fig. 3a–f).

**Fig. 4:**
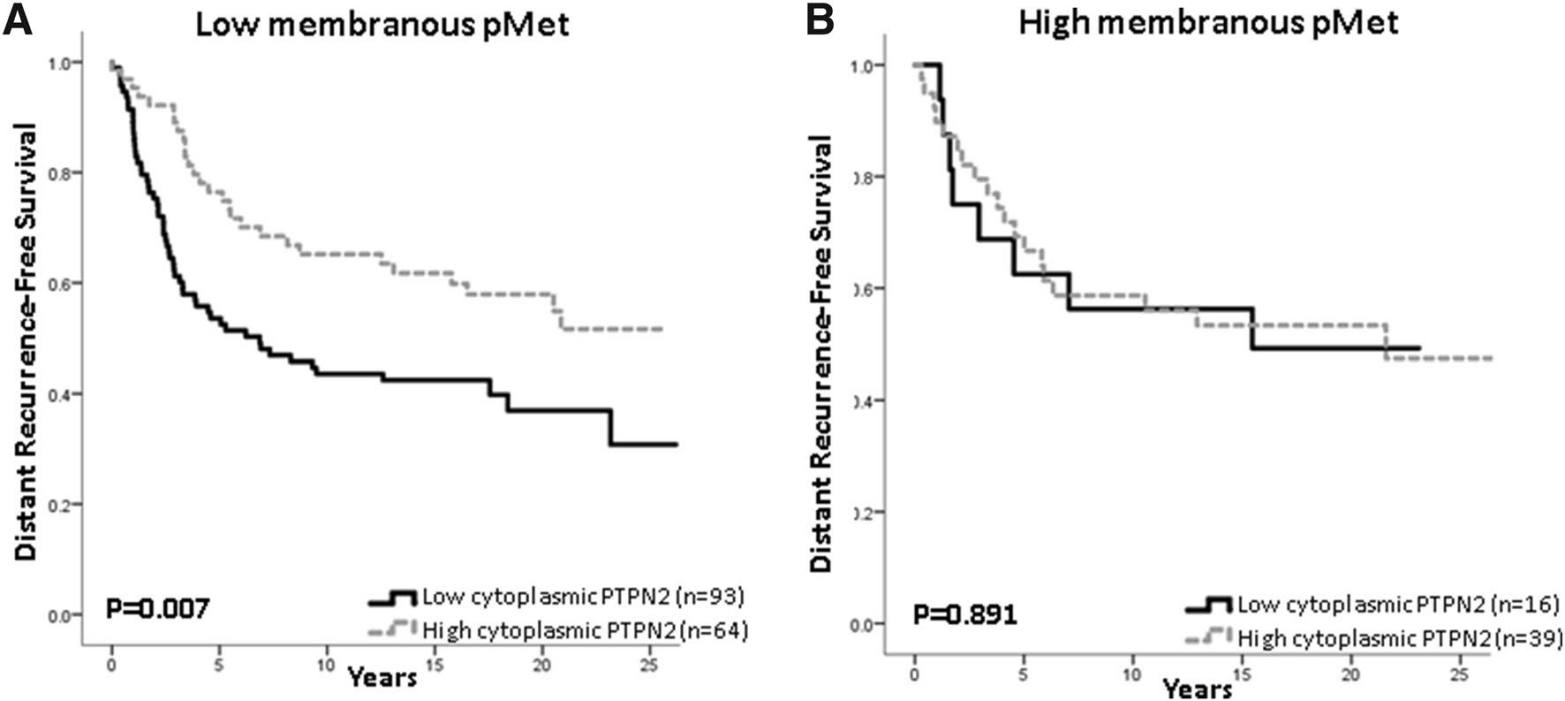
Distant recurrence-free survival in relation to PTPN2 protein expression and either low membranous pMet expression, **a** HR = 0.537; 95% CI 0.340–0.847, *p* = 0.008, or high-membranous pMet expression, **b** HR = 0.944; 95% CI 0.413–2.16, *p* = 0.891

Patients with tumours harbouring both low PTPN2 expression and low membranous pMet expression had a worse DRFS compared with patients with high PTPN2 expression and low pMet (Fig. 4a). For patients with tumours carrying high membranous pMet expression, there was no difference in survival in relation to PTPN2 protein expression (Fig. 4b). In the case of low membranous pMet, similar results were seen when selecting patients for Luminal A disease (HR = 0.491; 95% CI 0.258–0.935, *p* = 0.030) and HER2 overexpression (HR = 0.201; 95% CI 0.044–0.922, *p* = 0.039), but not for TNBC (HR = 0.541; 95% CI 0.190–1.541, *p* = 0.25).

### Prediction of radiotherapy benefit

**Fig. 5:**
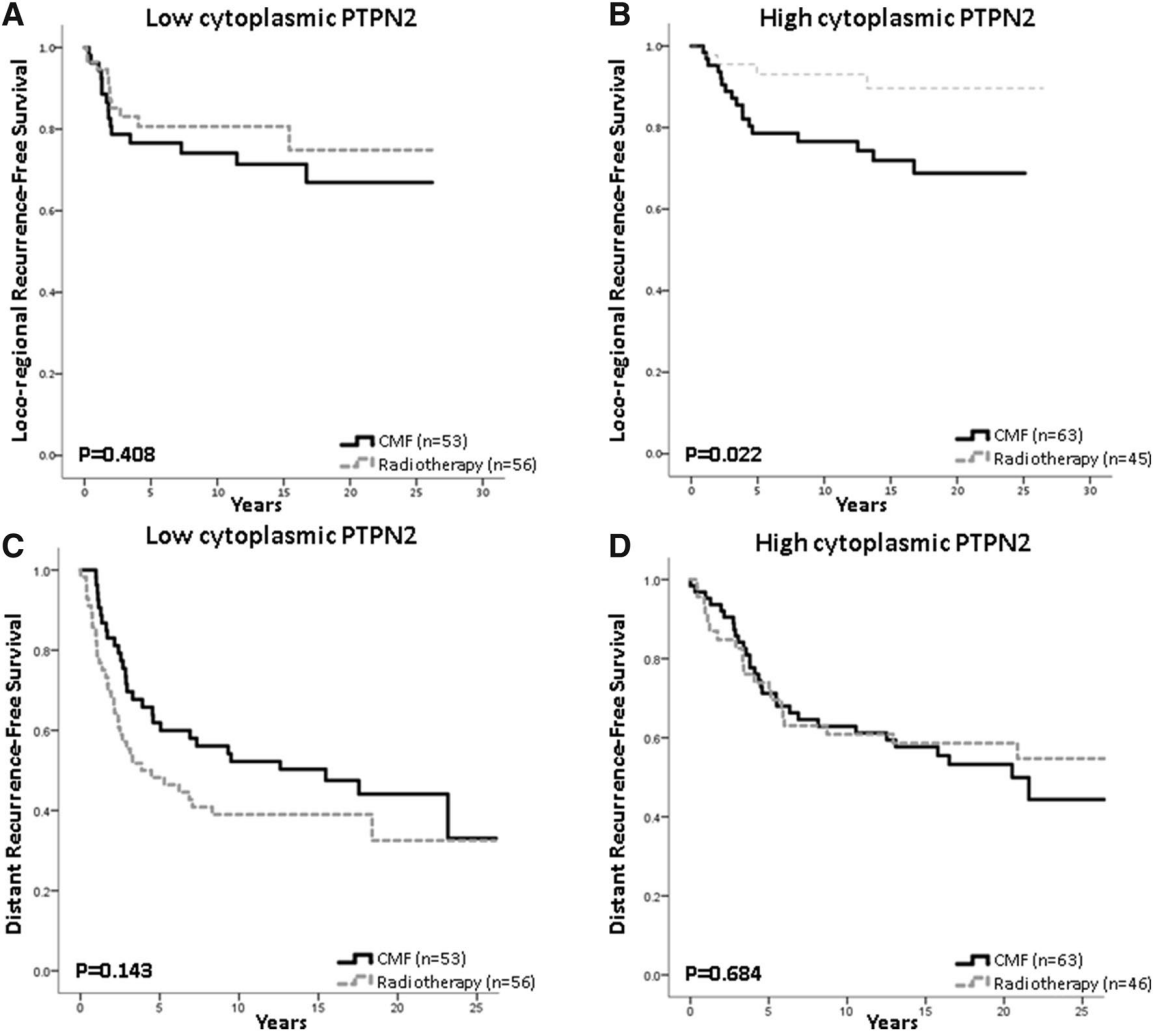
Estimated survival for patients treated with radiotherapy compared with chemotherapy in relation to PTPN2 protein expression. Loco-regional recurrence-free survival is shown for patients with tumours with **a** low PTPN2 expression (HR = 0.721; 95% CI 0.330–1.572, *p* = 0.410) and **b** high protein expression (HR = 0.301; 95% CI 0.101–0.896, *p* = 0.031), test for interaction: *p* = 0.20. Distant recurrence-free survival is shown for patients with tumours harbouring **c** low PTPN2 expression (HR = 1.44; 95% CI 0.881–2.63, *p* = 0.145) and **d** high protein expression (HR = 0.889; 95% CI 0.504–1.568, *p* = 0.685), test for interaction: *p* = 0.701

Radiotherapy is known to markedly reduce loco-regional recurrence risk and this was significant for patients with tumours expressing high cytoplasmic PTPN2 levels, but for patients with low cytoplasmic PTPN2 disease there was no significant difference in local recurrence rate between the radiotherapy and CMF groups (Fig. 5a, b). For patients with low, respectively, high cytoplasmic PTPN2 disease, distant recurrence-free survival was not significantly different between patients in the two treatment arms (Fig. 5c, d).

### PTPN2 knockdown responses are subtype-dependent

**Fig. 6:**
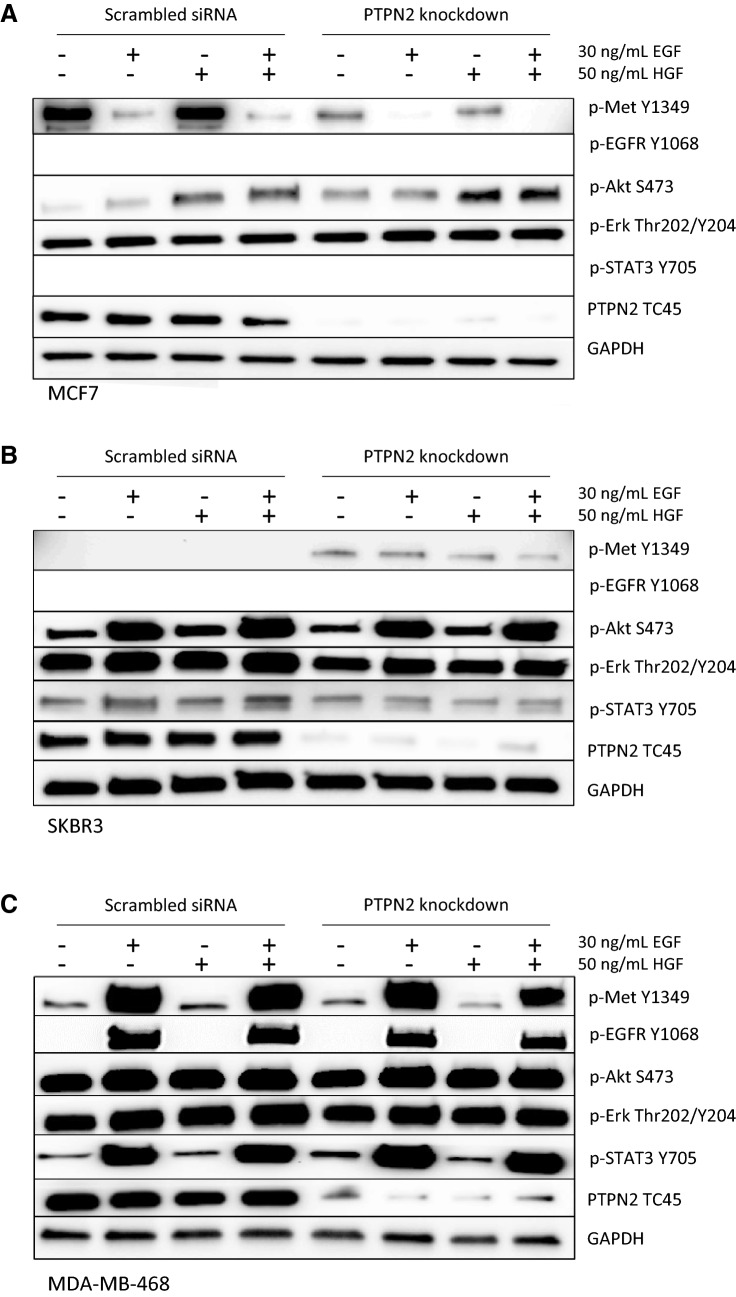
Effect of *PTPN2* knockdown and EGF/HGF stimulation on expression of proteins related to PTPN2. Cells were transfected with 10 nM scrambled siRNA or *PTPN2* siRNA for 48 h and serum-starved 24 h upon 30-min 30 ng/mL EGF and/or 50 ng/mL HGF treatment. The panels show representative images of Western blots; the experiments were repeated three times. GAPDH was used as loading control. Empty rows demonstrate that no protein was detected with the antibody. **a** MCF7 cell lysate, **b** SKBR3 cell lysate, **c** MDA-MB-468 cell lysate

To evaluate if the role of PTPN2 is subtype-dependent, the protein was knocked down in three different cell lines, representing three different subtypes. Three breast cancer cell lines were transfected for 48 h with siRNA targeting *PTPN2* or with scrambled siRNA. After 24-h starvation, cells were treated with EGF and/or HGF to stimulate pathway signalling. The immunoblots show that PTPN2 knockdown had different effects on the Luminal A representing cell line MCF7, the HER2-positive cell line SKBR3, and the TNBC cell line MDA-MB-468. PTPN2 depletion in MCF7 inhibited both Met constitutive phosphorylation and HGF-mediated phosphorylation and promoted Akt phosphorylation, both constitutive and HGF-mediated. However, pErk, a key protein in the Ras/MAPK pathway, was not affected by PTPN2 knockdown. pSTAT3 and pEGFR were not detected in MCF7 (Fig. 6a). In contrast, SKBR3 cells revealed increased Met phosphorylation levels upon PTPN2 knockdown. Here, PTPN2 loss did not affect Akt phosphorylation, though it did negatively affect pErk, both basic expression and EGF-mediated, and EGF-mediated STAT3 phosphorylation (Fig. 6b). PTPN2 loss did not affect phosphorylation of any of the tested proteins in the TNBC cell line MDA-MB-468 (Fig. 6c).

## Discussion

PTPN2 has been proposed to have a suppressive role in cancer. In this study, it was aimed to explore the role of PTPN2 loss in breast cancer. Low protein expression was found in 50.2% (110/219) of the tumours. Staining was found to be primarily cytoplasmic, nuclear staining was rarely detected. This is in line with previous findings in our lab, where 53.3% (354/664) low primarily cytoplasmic PTPN2 expression was found in a large cohort consisting of women with low-risk post-menopausal breast cancer (Karlsson et al. 2018). Gene copy number variation was analysed by ddPCR and it was found that the *PTPN2* gene is lost in 15.4% (33/214) of the cases. Few studies have been conducted on *PTPN2* gene copy number in cancer, we have previously reported similar numbers; 15.8% (34/215) copy loss in tumours from the post-menopausal patients of the Stockholm breast cancer trial (Karlsson et al. 2015) and 17.8% (26/146) in the aforementioned low-risk cohort (Karlsson et al. 2018).

We have previously shown that PTPN2 can predict tamoxifen therapy benefit in post-menopausal breast cancer patients (Karlsson et al. 2015, 2018). Here, we found involvement of PTPN2 in radiotherapy response. While it is widely known that radiotherapy significantly reduces the risk for loco-regional recurrence, this was not found significant for patients with low PTPN2 protein expression; it was true for patients with high PTPN2 expression. PTPN2 low protein expression and copy loss were related to a higher risk of distant recurrence in NHG grade 1 or 2 tumours. This has earlier been shown in a previous study where protein loss was associated with worse survival in NHG 1 tumours (Karlsson et al. 2018), indicating that PTPN2 is not prognostic in high-grade tumours, merely in low-grade tumours. Moreover, low PTPN2 protein expression was related to a higher relapse rate. This has previously been demonstrated with post-menopausal patients in this trial (Karlsson et al. 2015). While in the present study no direct association was found between PTPN2 and subtypes, PTPN2 low protein expression was correlated with a negative ER status, which is per definition non-Luminal and related to more aggressive disease compared with ER-positive tumours. As demonstrated by survival analysis in different subtypes in relation to protein expression, the prognostic value of PTPN2 seemed subtype-related. Although PTPN2 protein was commonly lost in TNBC, this was not significantly more compared with other subtypes, unlike shown in a previous report by Shields et al. (2013) showing 67% protein loss in TNBC tumours. However, it should be noted that their study had considerably fewer patients. The survival analysis in TNBC patients in relation to PTPN2 protein expression showed that loss of protein was not correlated with survival. In vitro studies showing that knockdown of PTPN2 did not alter phosphorylation levels of the tested proteins further indicated the lack of a significance of PTPN2 in triple-negative disease.

PTPN2 protein loss was correlated with less pAkt in the cytoplasm and more in the nucleus. We have previously demonstrated a relation between PTPN2 and Akt in breast cancer, in low-risk breast cancer patients, PTPN2 protein loss was likewise associated with less pAkt in the cytoplasm and more in the nucleus (Karlsson et al. 2018). In our previous study with post-menopausal breast cancer patients, copy loss and low mRNA levels were related to strong pAkt levels, most evident in Luminal A tumours (Karlsson et al. 2015). The relation between the Luminal A subtype, PTPN2, and pAkt was also seen in the Luminal A representing cell line MCF7, where knockdown of PTPN2 lead to increased Akt phosphorylation, but not in the other cell lines. The relation between PTPN2 and Akt has been demonstrated in other cancer types as well, like skin cancer and lung cancer (Lee et al. 2017; Omerovic et al. 2010). The nature of this relationship and the mechanism behind it remain unknown. However, as Akt is not a substrate of PTPN2, the association must be indirect; many substrates of PTPN2 are tyrosine kinases that can activate Akt.

Met has been previously found to be related to PTPN2 and suggested to be a substrate (Sangwan et al. 2008). Here, low PTPN2 protein expression was related to low expression of phosphorylated Met in the cytoplasm and membrane. Patients with low membranous pMet in their tumours and low PTPN2 expression had a poor survival rate, which was also seen in Luminal A disease and patients with tumours overexpressing HER2. In the ER-negative and HER2-positive cell line SKBR3, it was shown that Met phosphorylation increased after knockdown of PTPN2, further suggesting Met to be a substrate of PTPN2. Met phosphorylation has previously been shown to increase upon PTPN2 depletion and HGF stimulation in the ER-negative cell line HeLa (Sangwan et al. 2008). However, Met expression was found to be decreased upon PTPN2 knockdown in the Luminal A cell line MCF7. This suggests that the increase in Akt phosphorylation in PTPN2-depleted cells is not due to Met, but another mechanism. In HeLa cells, PTPN2-deficiency did not alter pAkt or pErk, however, knockdown was followed by increased STAT3 phosphorylation after EGF stimulation (Shields et al. 2013). PTPN2 has been previously shown to negatively regulate STAT3 (ten Hoeve et al. 2002). Counterintuitively, here, phosphorylation of STAT3 decreased upon PTPN2 knockdown in SKBR3 cells, though Akt and Erk phosphorylation levels remained unaltered.

In summary, the results presented here confirm previous studies demonstrating low PTPN2 protein expression in half of the breast cancer cases and copy loss in 15–18% of the cases. Furthermore, this study generated the hypothesis that the prognostic value of PTPN2 might be subtype-related. While PTPN2 does not seem to play a prognostic role in TNBC, loss of the protein is associated with worse patient outcome in Luminal A and HER2+ disease. The role of PTPN2 appears different in these two subtypes. In MCF7, representing Luminal A disease, loss of PTPN2 results in Akt phosphorylation independent of Met, whilst in SKBR3, representing HER2+ disease, Akt remains unaffected by PTPN2 depletion and Met is activated instead. To test this hypothesis and assess the implications on breast cancer treatment, more functional studies are needed in multiple breast cancer cell lines.
